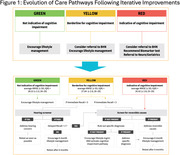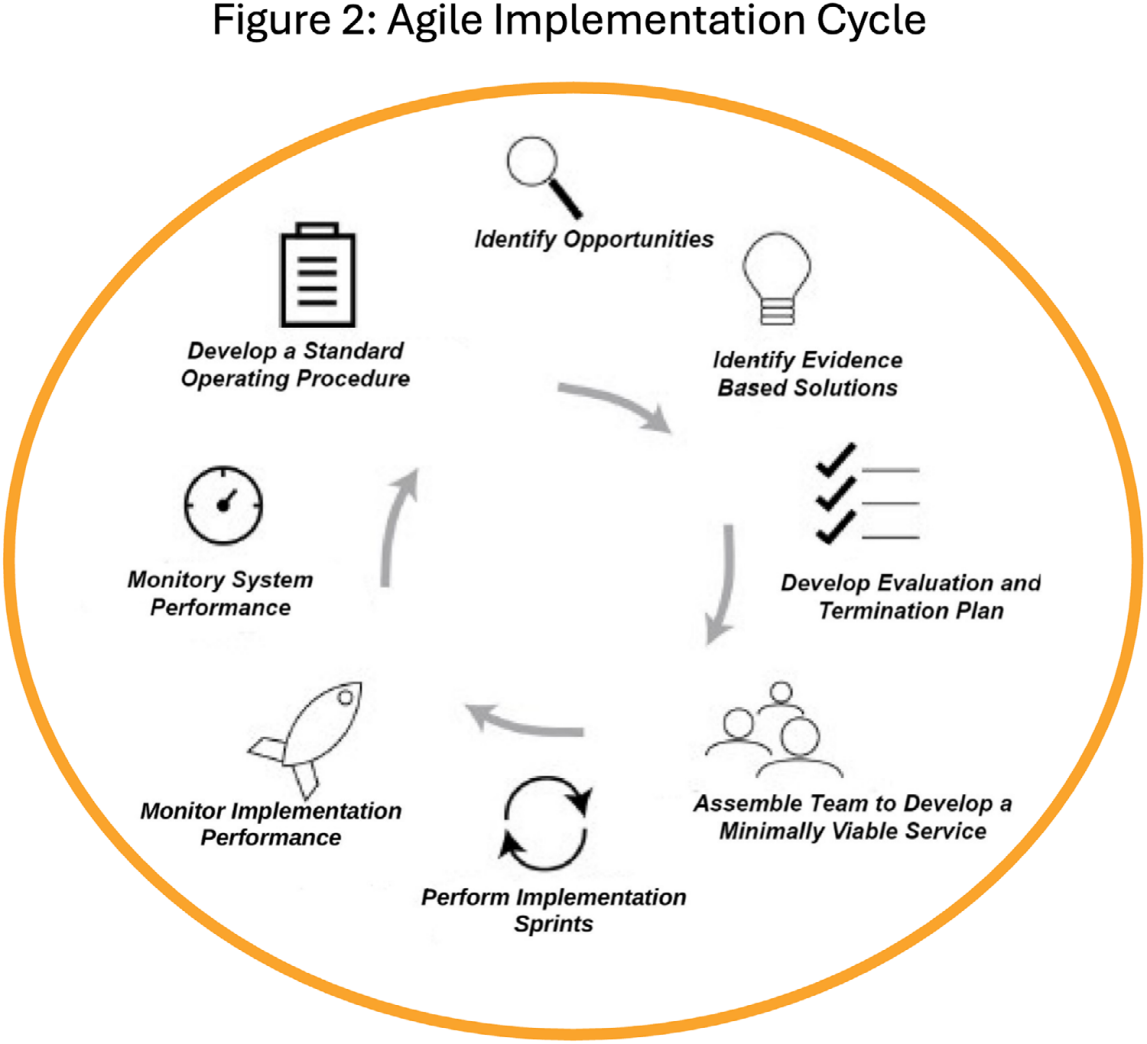# Optimizing primary care for cognitive impairment screening using agile implementation

**DOI:** 10.1002/alz.087555

**Published:** 2025-01-09

**Authors:** Diana Summanwar, Jared R. Brosch, Dustin B. Hammers, Nicole R Fowler, Deanna R Willis

**Affiliations:** ^1^ Indiana University School of Medicine, Indianapolis, IN USA; ^2^ Regenstrief Institute, Indianapolis, IN USA; ^3^ Indiana University Center for Aging Research, Indianapolis, IN USA

## Abstract

**Background:**

Screening for cognitive impairment in primary care faces challenges, including time constraints, provider apprehension, and limited diagnostic confidence. An effective initiative for improving screening must include strategies to foster behavioral change, and active provider engagement. Agile implementation science integrates findings from behavioral economics, complexity science, and network science, to address these challenges by confirming the demand to solve the problem; local solution adaptation; and the iterative ‘sprints’, or tests of change, that are focused on execution. This study, which is part of the Davos Alzheimer’s Collaborative (DAC) Early Detection Health System Preparedness Flagship program, explored workflows to support Digital Cognitive Assessment (DCA) in primary care, enhancing early detection of mild cognitive impairment (MCI) and dementia.

**Methods:**

Between June 1, 2022, and May 31, 2023, seven diverse primary care clinics participated in the DAC program. The initiative’s core was the integration of offering and performing Linus Health Core Cognitive Evaluation Digital Cognitive Assessment (DCA) for patients aged 65 and above. The selection of the digital screening tool, process workflows, and improvement cycles were co‐designed by the primary care providers, clinic staff, the Patient Advisory Council, and the implementation team using Agile Implementation. A Brain Health Navigator (BHN) role was designed to fill workflow gaps in primary care evaluation of abnormal screening and facilitate specialty care transition for patients needing referral.

**Results:**

Among the seven sites, five sites engaged in agile implementation and had similar performances, with an increase in DCA completion observed. A total of 1808 DCA screenings were performed on 1722 unique patients. The agile implementation process facilitated clinic‐specific adaptations, which resulted in an increase in the overall number of eligible patients completing the DCA screening.

**Conclusions:**

The adoption of an agile implementation process increased DCA screening uptake in primary care settings. The integration of a BHN and streamlined workflows proved crucial in enhancing the screening, diagnosis, and referral journey. This integration aligns with the principles of person‐centered care and facilitates service coordination. It also supports workforce initiatives and advances the field of health services research, ensuring that each step in the patient’s journey is both effective and efficient.